# INFLUENCES OF FAMILY AND HOUSEHOLD CHARACTERISTICS ON CHILDREN’S LEVEL OF PHYSICAL ACTIVITY DURING SOCIAL DISTANCING DUE TO COVID-19 IN BRAZIL

**DOI:** 10.1590/1984-0462/2021/39/2020297

**Published:** 2020-12-07

**Authors:** Cristhina Bonilha Huster Siegle, André Pombo, Carlos Luz, Luis Paulo Rodrigues, Rita Cordovil, Cristina dos Santos Cardoso de Sá

**Affiliations:** a Universidade Federal de São Paulo, Santos, SP, Brazil.; b Universidade de Lisboa, Cruz-Quebrada, Portugal.; c Escola Superior de Educação, Instituto Politécnico de Lisboa, Interdisciplinary Center for Educational Studies, Lisbon, Portugal.; d Escola Superior Desporto e Lazer de Melgaço, Research Center in Sports Sciences, Health Sciences and Human Development, Instituto Politécnico de Viana do Castelo, Viana do Castelo, Portugal.; e Interdisciplinary Center for the Study on Human Performance, Universidade de Lisboa Cruz-Quebrada, Portugal.

**Keywords:** COVID-19, Child, Motor activity, Child development, Quarantine, Pandemics, COVID-19, Criança, Atividade motora, Desenvolvimento infantil, Quarentena, Pandemia

## Abstract

**Objective:**

To evaluate if the variables child’s sex, age, presence of siblings, parents working remotely, and external space affect the level of physical activity (PA) of Brazilian children during social distancing imposed by the COVID-19 pandemic.

**Methods:**

An online questionnaire was applied by the LimeSurvey software from March to April 2020. Children were divided into four age groups, and the questionnaire comprised questions on family and household characteristics, domestic and children’s routines in the period of Brazilian social distancing. Based on the answers concerning children’s activities, the following variable was created: percentage of physical activity (%PA) in one-day period. Analysis of variance and regression analysis were performed to investigate the effect of demographic and parental activities on %PA.

**Results:**

The %PA decreases with increasing age, but increases with the availability of external space at home. No significant or interaction effects were observed for other variables. Age and external space at home are predictors of %PA.

**Conclusions:**

Household and personal characteristics of Brazilian children influence the level of physical activity performed by them during social distancing. Preventive measures can be adopted in the face of another similar period.

## RESUMO

**Objetivo:**

Investigar se as variáveis sexo da criança, idade, presença de irmãos, pais trabalhando de maneira remota e espaço externo afetam o nível de atividade física das crianças brasileiras durante o distanciamento social por COVID-19.

**Métodos:**

Aplicou-se questionário *online* pelo LimeSurvey, em março e abril de 2020, sobre características familiares e habitacionais, rotinas domésticas e infantis no período de distanciamento social brasileiro, sendo as crianças divididas em quatro grupos etários. Com base nas atividades infantis realizadas, criou-se a porcentagem de atividade física (%AF) em relação ao período de um dia e foi desenvolvida análise de variância para investigar o efeito das variáveis na %AF, além de análise de regressão.

**Resultados:**

A %AF diminui conforme o aumento da idade, mas aumenta com a disponibilidade de espaço externo no domicílio. Não houve efeito significativo para as demais variáveis, tampouco efeito de interação. A idade e o espaço externo ao domicílio são preditores da %AF.

**Conclusões:**

Características habitacionais e pessoais das crianças brasileiras influenciam o nível de atividade física realizada por elas durante o distanciamento social. Medidas preventivas podem ser empregadas caso haja necessidade de outro período semelhante.

## INTRODUCTION

In March 2020, the World Health Organization (WHO) classified the coronavirus outbreak as a global epidemic of COVID-19 disease.^1^ The main involvement of the pathology is respiratory, with acute respiratory distress syndrome, in which the virus spreads through respiratory droplets and contact.^1^ In mid-July 2020, 12,230,250 people had a confirmed diagnosis of the disease, and 554,290 people died from it. Of these cases, 1,755,779 were confirmed in Brazil, with 69,184 deaths in the territory - consisting in the second most affected country in the world.^2^ For adequate control of the spread of the pandemic, measures of social distancing and guidelines for staying at home have been globally adopted.^3^

Adults started working remotely from home or had their activities suspended. Around the world, schools also had their classroom activities suspended and children remained at home in social distancing.^4^ In Brazil, this measure was implemented throughout the national territory. Lockdown can have negative effects on the child population, considering that the school environment establishes routines for children in terms of nutrition, physical activity (PA), sleep, and other activities, and the absence from this environment can lead to a decrease in physical fitness and increase in body mass.^3^ ^,^ ^4^ In addition, staying at home can lead to a decrease in recreational and sporting activities and an increase in the time spent on screens (cell phones, televisions, computers, and tablet computers), which can negatively impact PA and increase the sedentary lifestyle of this population.^5^

Children with adequate PA levels have better cardiometabolic, musculoskeletal, mental, and cognitive health, better bone mineral density and motor performance, in addition to better self-esteem and self-image.^1^ ^,^ ^5^ ^,^ ^6^ Furthermore, physical inactivity leads to the development of obesity, other chronic diseases, and cardiovascular risks for young and older children^6^ ^,^ ^7^

A previous study^8^ identified changes in the habits of Brazilian children during the first month of social distancing. There was a decrease in the level of PA and an increase in sleep and screen times compared with the period prior to the social distancing measure. This period was detrimental to the children’s PA levels, and the sedentary time increased with age.^8^

There is the possibility of a second COVID-19 pandemic wave.^5^ Therefore, it is important to identify the household and family factors that influence the children’s PA level, in such a way to adopt preventive measures against sedentary lifestyle in this population. Thus, this study aimed to investigate whether variables, such as the child’s sex, age, presence of siblings, parents remotely working from home, and external space, affected the level of PA of Brazilian children during social distancing due to COVID-19.

## METHOD

This is a study with descriptive cross-sectional design that integrates international research from Universidade de Lisboa (UL) to understand the behavior of children under 13 years of age during the social distancing period resulting from the COVID-19 pandemic.

To assess how families with children aged 0 to 12 years are dealing with lockdown due to the COVID-19 situation, a questionnaire was created on LimeSurvey, a free software for applying online questionnaires, which can use databases for data persistence, hosted at Faculdade de Motricidade Humana - UL. This questionnaire was prepared by a committee of specialists in the area, tested in 15 families (pretest), and disclosed after adjustments in the presentation of responses in relation to the number of hours of activities performed by children. The research was approved by the Research Ethics Committee of Universidade Federal de São Paulo (Certificate of Presentation for Ethical Consideration [CAAE] 30930120.2.000.5505, No. 0413/2020).

In Brazil, the questionnaire was launched online on March 24, 2020 and disseminated on social media (Facebook, Instagram, WhatsApp) and via e-mail, following the snowball sampling technique. It is anonymous, takes five minutes to complete, and comprises four sections:

• Family: family composition, number of children and adults who are at home, and how many of them are doing their professional activity or working from home.
- Household characteristics: type and characteristics of the house, whether or not there is indoor and outdoor space for PA.
- Domestic routines: level of concern in relation to the situation of COVID-19 and the way in which family routines are being adjusted (PA time, screen time, sleep, family activities).
- Children’s routines: characterization of each child (age, sex, health status) and hours spent in different activities in the previous day.

Questionnaires answered by parents/guardians of all children under 13 years of age living in the same residence, from March 25 to April 24, 2020 during social distancing, were included in this study, totalizing 1,352 responses. All participants read information about the study and agreed to the conditions, clicking to proceed on the first page of the survey. They could withdraw at any time, not continuing to fill in the questionnaire or sending information. After clearing the database, responses related to 816 children aged 0 to 12 years (410 boys, 403 girls, and three without identification) were included in this study, and responses from 536 children (39.6% of the 1,352 children initially reported) were excluded due to missing or obviously wrong information (for example, more than 24 hours reported in a day or no sleep time reported for children). Children were divided into four age groups:

• Group 1: 0-2 years (n=187).
- Group 2: 3-5 years (n=206).
- Group 3: 6-9 years (n=285).
- Group 4: 10-12 years (n=138).

Five categories of activities were analyzed:

1. Intellectual (school assignments and online classes).
2. Playtime spent on screens (games, movies, social media, Internet, video and voice calls).
3. Playtime without PA (reading, drawing, painting, board games, etc.).
4. Playtime with PA (running, jumping, playing hide-and-seek, etc.).
5. PA (planned, inside and outside the house, walking a dog).

Categories 1 to 3 were grouped to calculate sedentary time, and categories 4 and 5 were added to calculate general PA. These values were converted into a percentage of total time, called percentage of PA (%PA).

Descriptive statistics were performed for the initial characterization of 816 children divided by age groups, the conditions of their families, and household characteristics. Four different analyses of variance (ANOVA) were performed to investigate how variables related to children (sex and number of children in the family), household characteristics (existence and dimension of external space), and the adults’ work situation (whether all the adults are remotely working from home or not) affected the %PA by age group (groups 1 to 4). A regression analysis (forward stepwise regression) was developed to investigate the variables that better predicted %PA performed by children. The age of the children and the variables evaluated by ANOVA were inserted in the model. Qualitative variables (sex, external space, and working from home) were converted into dummy variables before being inserted in the regression, and the pairwise deletion method was employed in the regression to accommodate missing values. The analyses were performed using the *Statistical Package for the Social Sciences* (SPSS), version 23, with a significance level of 0.05 as reference.

## RESULTS

Descriptive data of %PA and results of analysis of variance according to sex, external space, number of children at home, and parents’ remote work, according to the age group, are shown in Table 1. There is a main effect of age groups on all variables: the %PA of children decreased as the age group increased (Table 1).

**Table 1:** Effects of sex, availability of external space, number of children at home, and adults working from home on the percentage of children’s physical activity, according to parents and guardians.

		Percentage of daily physical activity (%)				Two-way ANOVA
		Age group				
		0-2 years	3-5 years	6-9 years	10-12 years	
		Mean±SD	Mean±SD	Mean±SD	Mean±SD	
Sex	Boys	10.3±16.2	6.2±8.9	4.9±7.9	4.9±10.2	F_age_ (3, 788)=7.25; p<0.001* F_sex_ (1, 788)=2.17; p=0.140 F_age*sex_ (3, 788)=1.17; p=0.319
	Girls	6.3±12.9	6.6±8.8	3.9±5.7	4.5±6.9	
Available external space	Unavailable	9.8±19.4	3.6±6.6	1.9±3.8	4.9±13.3	F_age_ (3, 787)=9.75; p<0.001* F_space_ (2, 787)=10.11; p<0.001* F_age*space_ (6, 787)=1.92; p=0.074
	Small	6.7±10.8	6.6±7.9	4.8±7.4	3.7±6.1	
	Large	12.8±17.7	10.6±12.6	6.8±8.1	6.7±8.2	
Number of children	Only one	9.2±16.5	6.6±9.5	4.2±6.8	3.7±6.2	F_age_ (3, 790)=6.932; p<0.001* F_children_ (1, 790)=0.005; p=0.944 F_age*children_ (3, 790)=0.700; p=0.552
	From 2 to 5 children	7.7±12.1	6.3±8.4	4.5±7.1	5.5±10.3	
Adults remotely working^&^	All of them At least one exempt from it	6.8±10.9 9.7±16.5	6.3±9.3 6.6±8.7	3.4±5.5 5.0±7.7	4.1±6.5 5.0±9.7	F_age (_3, 791)=6.40; p<0.001* F_work_ (1, 791)=3.46; p=0.063 F_age*work_ (3, 791)=0.586; p=0.625

Differences in %PA regarding the tested variables demonstrated major effects on the availability of external space and the number of children, but not significant for the child’s sex, number of children at home, and number of adults remotely working from home (Table 1). There was no interaction effect between the analyzed variables (Table 1). Thus, children living in houses with large external space (greater than 12 m^2^) presented higher %PA than children in houses without external space (p<0.001) or small external space (p=0.001). There was no significant difference in the percentage of physical activity performed by children without external space and with small external space (up to 12 m^2^) (p=0.225).

Regarding the child’s sex, no significant differences were found between the %PA of boys and girls (Table 1). In addition, and although there is no main effect on the number of children, there is an inversion in %PA over the age groups. Thus, in the first two age groups, those who were only children had a higher %PA; the opposite was verified in the two older age groups, considering that higher %PA values were found in the cases with more children living in the house.

Although there is no significant difference in the %PA of children who had at least one adult available and exempt from remote work compared with those who were at home with all adults remotely working, a trend towards a higher %PA of children when there was an adult exempt from remote work was observed.

According to the linear regression analysis (Table 2), age and type of spaces were significant predictors of children in relation to %PA, justifying 4.7% of the overall variation (Table 2). When observing the predictive effect of each independent variable, age was verified as a predictor, suggesting a decrease of 1.5% in the %PA for each age. In addition, having an external space larger than 12 m^2^ was also a predictive factor, with a 4.2% increase in %PA (Table 2).

**Table 2:** Linear regression, summary of the predictor variables for percentage of physical activity.

Predictor variables	β	Beta	T	p-value	F	R^2^
Age	-1.5	-0.157	-4.415	<0.001	18.754	0.047
Large external space	4.269	0.161	-4.512	<0.001		

## DISCUSSION

The results show that having large external space at home and age influence the %PA performed by children during social distancing due to COVID-19. According to recommendations of the WHO, children should do at least one hour a day of PA of moderate to vigorous intensity, and PA is considered as the body movement produced by skeletal muscles with demand for energy expenditure.^9^ Although means of %PA performed by children in most cases exceed the recommended time, there was no control over the intensity of this activity, in such a way that is not possible to state that this population is following the recommendation. In addition, a previous study showed a decrease in the performance of PA by Brazilian children during social distancing when compared with the period prior to this measure.^8^ These factors demonstrate the importance of monitoring the PA practiced by children in this period, especially among older children.

A study on Italian children also found a decrease in the time spent in sports, and increase in times for sleep, screen, and feeding during social distancing, generating increased risk factors for obesity.^4^ This was also verified in a Canadian child population, which had an increase in sedentary behavior, screen time, and decreased PA, with lower PA in older children and girls.^1^ A Canadian research identified that children who live in houses and who have a dog had a higher level of external PA compared with those who live in apartments, considering that this investigation also evaluated external PA such as walking and cycling.^1^ Results of the present study corroborate the decrease in PA according to age, but not according to sex. Increase in sedentary behavior was also observed during social distancing among Chinese children.^7^

A Brazilian study on preschool children aged 4 to 6 years during the school term (without the need for social distancing) demonstrated that the availability of physical space outdoors for games and playful activities at the place of residence influences the level of PA.^6^ The results of the present study agree with the aforementioned research, demonstrating that children who live in houses with external environments larger than 12 m^2^ perform a higher level of PA. Thus, the relevance of this variable is noteworthy in moments prior to and during the pandemic. Small external spaces may limit children’s PA,^10^ and the children’s stay at home also restricts their external activities and interaction with other children, which have a psychosocial impact, generating stressful responses that can affect behavior, memory, cognition, and attention. ^3^ Moments spent in outdoor activities and in nature are critical to generate healthy movement behaviors, better sleep habit, and less sedentary behavior.^1^

Studies show that girls are naturally less active than boys^10^ and that, during the Canadian social distancing, boys were more likely to reach the recommended PA levels than girls;^5^ however, the results of the present study do not show differences in %PA between sexes. The Brazilian social distancing may have been similarly affecting boys and girls. According to a Brazilian study, there was no difference between the levels of PA of boys and girls in periods without social distancing.^6^ Cultural and regional characteristics may be associated with differences found in the research.

Barros et al.^6^ identified in their study that families with two or more children in regular school term are 50% less likely to have a low level of PA, assuming that siblings would have more interaction in games and playful activities. Hesketh et al. also observed lower levels of PA in only children;^11^ nevertheless, the findings of the present study demonstrated that, during the pandemic period, the presence of another child in the Brazilian household did not influence the %PA. With school activities being remotely carried out at home and with siblings of different age groups, older siblings are assumed to be more involved in remote activities with school assignments, whereas younger ones may not have as much assignments as they do, in such a way there is neither this interaction nor the influence of the number of children in the family.

Hence, older children may face a greater demand for school assignments, which also explains the lower %PA with increasing age. A systematic review found that the level of PA decreases with increasing age and is related to the evolution from primary to secondary school.^12^ In addition, smaller spaces and adequate toys favor the PA of younger children,^10^ whereas older children need larger and external spaces for performing more activities.

According to the results of this study, there is no higher %PA in children living in Brazilian houses with at least one guardian who is exempt from remote work. A study conducted before social distancing reports that the time spent at work was deemed as lack of time to stimulate the child,^10^ but the pandemic brought a new reality. There is a context of restriction of family movement, with emotional and psychological burden of guardians or parents, accumulation of work done from home, and concerns about an uncertain period, impacting the child’s support system and care practices.^13^ ^,^ ^14^ The closure of schools brought new forms of stress to those responsible for providing care and changed the usual care practices, requiring parents to assume greater responsibilities and to have more time for home education.^13^ ^,^ ^14^ In addition, 83% of children under the age of 4 are primarily taken care by women in their homes,^13^ and staying at home also generated other social changes such as the increase in domestic violence.^13^ The results of this study indicate that adults being exempt from remote work does not necessarily mean that they will be more available and able to stimulate the child’s PA.

In a period prior to the pandemic, families living in apartments have already been concerned about not causing discomfort to neighbors due to noises from children’s activities.^10^ This concern may be greater during the period of social distancing. With all residents staying at their homes full-time, there was an increase in noise complaints, including noises from children playing and carrying out other activities.^15^ In order to follow the standards of good coexistence and avoid possible penalties for excessive noise, according to Brazilian Federal Law No. 3,688,^16^ families who live in apartments may be limiting the noise of their children, hence reducing their physical and playful activities within homes without large external spaces. This hypothesis is related to the results found in the present study regarding the availability of external space, number of children, and sex, considering that the restriction of noise and activities in houses without large external spaces would be equal for boys and girls.

A Canadian study showed that the parental ability to restrict the children’s screen time during the day was the greatest predictor in achieving Canadian guidelines for movement behavior during social distancing.^5^ Although this variable was not evaluated in the present investigation, such limitation is deemed important, considering that children may be substituting PA time for screen time, which is common nowadays.^10^

PA can be beneficial in preventing and treating depressive symptoms and mental disorders in children and adults, who are more susceptible to such health issues during this period.^1^ ^,^ ^3^ ^,^ ^17^ In addition, maintaining an adequate level of PA can help reduce the risk of respiratory infections and improve the immune function and vaccine effectiveness.^17^ ^,^ ^18^ Therefore, physical exercise can assist in coping with this period,^1^ ^,^ ^17^ but, as aforementioned, there was a reduction in children’s PA levels due to the lockdown.^8^ The school provides PA at different times.^6^ ^,^ ^19^ Extracurricular activities and sports in public places, such as parks, are also hampered by social distancing.^13^ Studies on PA conducted during this pandemic are important for global public health^17^ and for measures related to healthcare prevention and promotion to be adopted in future periods of social distancing - such as telemonitoring and intervention programs, guidance and education for guardians or parents concerning the importance of PA in childhood.^3^ ^,^ ^4^ ^,^ ^6^ ^,^ ^17^ Furthermore, alternative activities for small spaces should be encouraged and taught to the families.

The lack of information for characterizing the sample per Brazilian regions and the non-identification of the socioeconomic level consist in limitations of the study. The %PA of Brazilian children can vary depending on regions due to cultural characteristics and the fact that Brazilian States have made different decisions regarding social distancing, some with greater flexibility and others that imposed lockdown for the cities. The fact that this is a cross-sectional study also makes the research susceptible to bias.

Household characteristics, such as dimension of external space, and personal characteristics, such as age, influence the level of PA of Brazilian children in social distancing due to COVID-19. Preventive and educational initiatives may be necessary to mitigate the impact on children’s physical activity in future periods of social distancing.
